# The *Pseudomonas aeruginosa* ExoY phenotype of high-copy-number recombinants is not detectable in natural isolates

**DOI:** 10.1098/rsob.170250

**Published:** 2018-01-31

**Authors:** Antje Munder, Justin Rothschuh, Bastian Schirmer, Jens Klockgether, Volkhard Kaever, Burkhard Tümmler, Roland Seifert, Christina Kloth

**Affiliations:** 1 Clinic for Pediatric Pneumology, Allergology and Neonatology, Hannover Medical School, Carl-Neuberg-Strasse 1, 30625 Hannover, Germany; 2 Institute of Pharmacology, Hannover Medical School, Carl-Neuberg-Strasse 1, 30625 Hannover, Germany; 3 Institute of Functional and Applied Anatomy, Hannover Medical School, Carl-Neuberg-Strasse 1, 30625 Hannover, Germany; 4 Research Core Unit Metabolomics, Hannover Medical School, 30625 Hannover, Germany; 5 Biomedical Research in Endstage and Obstructive Lung Disease (BREATH), German Center for Lung Research, Hannover, Germany

**Keywords:** *Pseudomonas aeruginosa*, type III secretion system, ExoY, lung infection

## Abstract

The nucleotidyl cyclase ExoY is an effector protein of the type III secretion system of *Pseudomonas aeruginosa*. We compared the cyclic nucleotide production and lung disease phenotypes caused by the ExoY-overexpressing strain PA103Δ*exoUexoT::Tc* pUCP*exoY*, its vector control strain PA103Δ*exoUexoT::Tc* pUCP18, its loss-of-function control PA103Δ*exoUexoT::Tc* pUCP*exoY* K81M and natural ExoY-positive and ExoY-negative isolates in a murine acute airway infection model. Only the *P. aeruginosa* carrier of the *exoY-*plasmid produced high levels of cUMP and caused the most severe course of infection. The pathology ascribed to ExoY from studies using the high-copy-number plasmid on mammalian cells *in vitro* and *in vivo* was not observed with natural *P. aeruginosa* isolates. This indicates that the role of ExoY during infection with real-life *P. aeruginosa* still needs to be resolved.

## 1. Introduction

The type III secretion system (T3SS) of *Pseudomonas aeruginosa* enables the bacterium to inject the T3SS-associated effector proteins ExoS, ExoT, ExoU and ExoY directly into host cells via a needle-like structure [1]. In most cases, functional expression of ExoS and ExoU is mutually exclusive [2]. Both ExoS and ExoT—sharing the highest homology out of the four known T3SS enzymes—exhibit ADP-ribosyltransferase activity, interfering with manifold signalling pathways in the host cell, such as the Ras-signal transduction [2–5]. By contrast, ExoU causes direct cytotoxic effects on host cells by its phospholipase A2 activity [6]. While ExoS, ExoT and ExoU are well established virulence factors of *P. aeruginosa*, little is known about the role of ExoY during *P. aeruginosa* infection.

The effector protein ExoY was originally described as an adenylyl cyclase with structural similarities to the bacterial cyclases CyaA from *Bordetella pertussis* and oedema factor (EF) from *Bacillus anthracis* [7], having no significant impact on cytotoxicity *in vitro*, which led to the persisting evaluation of the exotoxin as having no clinical relevance [8,9]. Contrary to this, several recent studies have been published on a *P. aeruginosa* mutant bearing an additional plasmid coding for *exoY* (PA103Δ*exoUexoT::Tc* pUCP*exoY*) [7]. In these studies, a distinct phenotype of cells or animals infected with the ExoY-overexpressing mutant could be demonstrated [10–13].

ExoY synthesizes numerous cNMPs [14–16]. cUMP turned out to be the most prominent cyclic nucleotide generated in the lungs of mice infected with ExoY expressing *P. aeruginosa* [15]. In the infected lungs, the accumulated cUMP leaked into the extracellular compartments [15], where it induced chemotaxis or metabolic responses. cUMP is known to be transported across the plasma membrane by multidrug resistance protein (MRP) 4/5 [15,17] which probably led to the appearance of cUMP in sera, urine and faeces of these infected mice [15].

ExoY is an obligatory effector of all T3SS in *P. aeruginosa*, but ExoS and ExoU are almost mutually exclusive so that the *P. aeruginosa* population is currently differentiated into a major ExoS-positive clade, a minor ExoU-positive clade and a minute T3SS-negative clade [18,19]. In this study, we compared the cNMP levels and lung disease of mice that were infected with T3SS-negative or T3SS-positive *P. aeruginosa* isolates [20,21] with those infected with the engineered *P. aeruginosa* PA103 strains PA103Δ*exoUexoT::Tc* pUCP*exoY*, PA103Δ*exoUexoT::Tc* pUCP*exoY* K81M and PA103Δ*exoUexoT::Tc* pUCP18 carrying plasmids with *exoY* wild-type sequence, the loss-of-function mutation K81M *exoY* and the empty plasmid, respectively*.* The latter three strains have been used in the literature to dissect the function of ExoY in the absence of other T3SS effectors, but the side-by-side comparison with natural isolates has not yet been performed although this direct comparison provides a clue about the physiological relevance of phenotypes generated by a recombinant strain carrying multiple copies of *exoY in trans*.

## 2. Material and methods

### 2.1. Cultivation of bacteria

Bacterial stocks (80% Luria Bertani (LB) broth/20% glycerol) were stored at −80°C. For experiments the recombinant *P. aeruginosa* strains PA103Δ*exoUexoT::Tc* pUCP*exoY* hereafter designated ‘ExoY’, PA103Δ*exoUexoT::Tc* pUCP*exoY* K81M designated ‘ExoY^K81M^’ and PA103Δ*exoUexoT::Tc* pUCP18 designated ‘ΔExoY’ [5] were streaked on Vogel Bonner medium plates containing 400 µg ml^−1^ carbenicillin and incubated at 37°C overnight. The next day, a large loopful of bacteria was suspended in PBS and the number of colony forming units (cfu) ml^−1^ determined by measuring the optical density with the UV-160A spectrophotometer, OD_540_ = 0.25 = 2×10^8^ cfu ml^−1^. The environmental isolates B420 and PT22 [20,21] were cultivated at 37°C in LB for 14 h, harvested by centrifugation, washed with PBS and adjusted to their final density extrapolated from a standard growth curve. The factors of dilution were calculated from growth curves that had been recorded in prior experiments. A short description of all bacterial strains used in the study is listed table 1.

**Table 1.** Bacterial strains.

strain	strain designation	source	virulence	T3SS-effectors	ExoY
PA103Δ*exoUexoT::Tc* pUCP*exoY*	ExoY	genetically engineered [7]	√√	—	√
PA103Δ*exoUexoT::Tc* pUCP*exoY* K81M	ExoY^K81M^	genetically engineered [7]	—	—	√ (loss-of-function mutation at position K81)
PA103Δ*exoUexoT::Tc* pUCP18 (vector control strain)	ΔExoY	genetically engineered [7]	—	—	—
B420	B420	river [20]	—	—	—
PT22	PT22	river [21]	√	√	√

### 2.2. DNA preparation

For preparation of genomic DNA, strains ExoY and ExoY^K81M^ were washed from VB plates containing carbenicillin in a total volume of 5 ml PBS and pelleted by centrifugation; 5 ml liquid cultures of strains B420 and PT22 were pelleted as well. DNA was then prepared from bacterial cells following standard procedures which had been optimized for Gram-negative bacteria [22].

### 2.3. ExoY real-time PCR

Multiwell PCR (StepOnePlus, Applied Biosystems) was performed with 1 ng genomic DNA per well, 50 nM primer solution (5′-GGA CGG ATT CTA TGG CAG GG-3′, 5′-CGT CGC TGT GGT GAA ACA TC-3′), 7 µl H_2_O and 10 µl Power SYBR Green PCR Master Mix (Life Technologies, Delhi, India). The *exoY* copy number was determined by comparison with a dilution series of the *exoY-*plasmid (pET16b*exoY*wt; Novagen/Merck KgaA, Darmstadt, Germany) and normalized to the Ct-value of the *hydrogen cyanide synthase subunit* (*hcnB*) gene located adjacent to *exoY* in the *P. aeruginosa* genome.

### 2.4. Murine airway infection model

Eight- to 10-week-old female C57BL/6 J mice (Janvier, Germany) were maintained in the animal facility of Hannover Medical School in microisolator cages with filter top lids at 21 ± 2°C, 50 ± 5% humidity and a 14 L : 10 D cycle. They were supplied with autoclaved, acidulated water and fed ad libitum with autoclaved standard diet. Prior to infection mice were anaesthetized (5 mg midazolam kg^−1^ and 100 mg ketamine kg^−1^) intraperitoneally and to reduce anaesthesia-induced salivation each animal received atropine (dose: 1 µg per animal) subcutaneously half an hour before. Bacteria were adjusted to 10^6^ cfu and in a volume of 50 µl PBS instilled intratracheally (i.t.) to the mice lungs as described previously [23]. For the determination of the actual dosage, serial inoculates were plated on LB agar plates. Mice were sacrificed by an overdose of anaesthetic 0–72 h post-infection. Blood was taken by puncture of the right heart ventricle and broncho-alveolar lavage (BAL) was performed using 1 ml PBS. Individual lung lobes were weighed and used for mass spectrometric analysis of cyclic nucleotides and for histology.

### 2.5. Disease score, temperature, body weight and lung score

During infection mice were monitored regularly for 72 h (4, 6, 8, 10, 12, 24, 48, 72 h) by rectal temperature and body weight. The overall health was assessed by a multiparametric disease score as described before [23]. In brief, vocalization, piloerection, posture, locomotion, breathing, curiosity, nasal secretion, grooming and dehydration were recorded and dysfunctions determined by 0, 1 or 2 points. Adding these points resulted in the following score: unaffected (0–1); slightly affected (2–4); moderately affected (5–7); severely affected (8–10); moribund (greater than or equal to 11). Inflammation in infected lungs was assessed using a semi quantitative pathohistological score. Shortly, lung histological changes were scored on a scale from 0 to 2 points (no pathologic alteration = 0, mild pathologic changes = 1, severe pathologic changes = 2). Points were given separately for macroscopic evaluation of the lung tissue (visual anomalies as haemorrhage, atelectasis, 0–2), thoracic bleeding (0–1) and BALF (content of blood, 0–2) and microscopic analyses of lung tissue (oedema, apoptosis and inflammatory influx, 0–2) yielding a sum score ranging from 0 to 7.

### 2.6. Histology

For histology, lungs from mice sacrificed 2, 12 and 72 h after infection, were fixed with 4% formalin (v/v) and embedded in paraffin. The paraffin blocks were cut into 4 mm slices and stained with haematoxylin/eosin (Merck, Darmstadt, Germany). Microphotographs were performed using a Zeiss AxioVert 200M microscope and a Zeiss Axio Scan.X1 scanner. Exemplarily, micrographs of each group are presented in figure 2*b* or *c*.

### 2.7. Mass spectrometry

Tissues (50–200 mg) were transferred to 2 ml FastPrep vials containing 200 mg garnet matrix and one ¼-inch ceramic sphere (lysing matrix A). Eight hundred microlitres of organic extraction solvent (70/30 ethanol/water [v/v] containing 12.5 ng ml^−1^ of the internal standard tenofovir) were added and tissue was homogenized using a FastPrep-24 system (MP Biomedicals, Santa Anna, CA) at a speed of 5 m s^−1^ for 60 s. Phosphodiesterases were inactivated by heating the homogenate for 15 min at 95°C. After centrifugation (20 800*g*, 10 min, 4°C), 600 µl of the supernatant fluid were dried at 40°C under a gentle nitrogen stream. The residual pellet was dissolved in 150 µl water and analysed by HPLC-MS/MS as described earlier [16,24–27]. Chromatographic data were collected and analysed with Analyst 1.5.1 software (ABSCIEX). The LLOQ for standard cAMP was 0.04 pmol per sample, for standard cGMP 0.07 pmol per sample, for standard cCMP 0.07 pmol per sample, and for standard cUMP 0.4 pmol per sample [25].

### 2.8. Statistics

Data are presented as means ± s.e.m. of *n* = 6 animals (animal studies) or based on three to four independent experiments performed in technical duplicates. GraphPad Prism 7.0 (San Diego, CA, USA) was used for calculation of means and s.e.m.

## 3. Results

### 3.1. *ExoY* copy numbers

**Figure 1.**
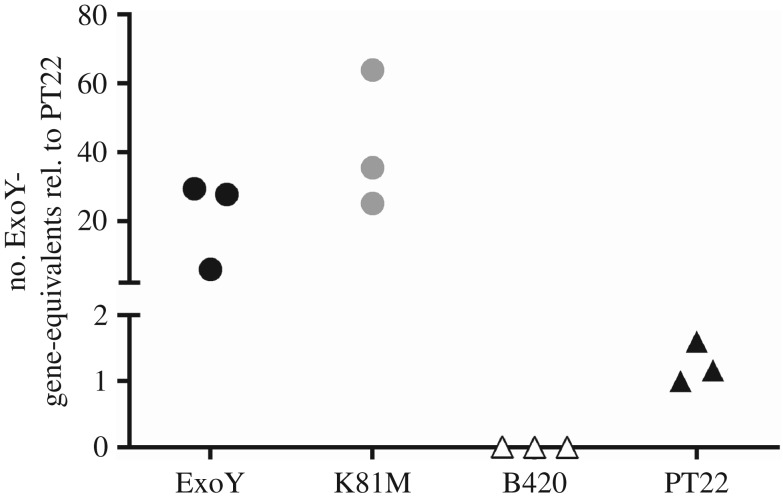
Copy number of *exoY* in *P. aeruginosa* B420, PT22 and the recombinant PA103 ExoY and K81M. Copy number of *exoY* (PA2191, PAO1 genome coordinates 2410344–2411480) was determined from three independent preparations of genomic DNA by real-time PCR and normalized to the signals of the first DNA preparation of strain PT22 and of the adjacent *hcnB* gene (PA2194, coordinates 2412857–2414251). For better discrimination of low and high copy numbers of *exoY* in natural and genetically engineered strains *y*-axis was interrupted.

We hypothesized that the discordant literature reports on ExoY-mediated phenotypes [8,9,11,13] may be ascribed to the different copy numbers of the *exoY* gene in natural *P. aeruginosa* isolates and the recombinant *P. aeruginosa* PA103 strain carrying *exoY* on a plasmid and deletions of the *exoU* and *exoT* T3SS effector genes on the chromosome. Quantitative real-time PCR revealed as expected single to two copies of *exoY* in the sequenced T3SS-positive *P. aeruginosa* strain PT22 [21] and no *exoY* signal in the T3SS-negative strain B420 [20] (figure 1). Conversely, the recombinant PA103 carriers of the *exoY*-plasmid were harbouring dozens of *exoY* genes in their cells whereby the plasmid copy number was higher for the functionless ExoY^K81M^ mutant than for the functional ExoY mutant (figure 1).

### 3.2. Acute murine *Pseudomonas aeruginosa* airway infection

**Figure 2.**
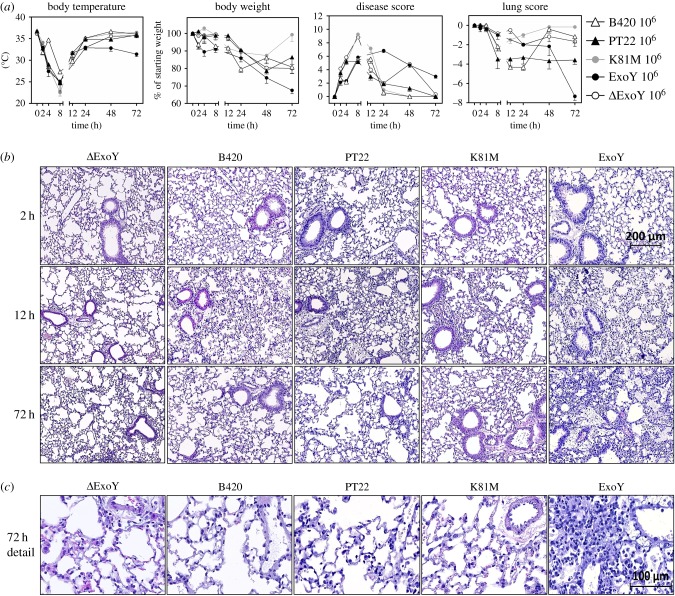
Phenotype of infected mice. (*a*) Decrease in body temperature and body weight, disease score and lung score of infected mice 0–72 h post-infection. Bars represent the mean ± s.e.m. of *n* = 6 animals after infection with 10^6^ cfu per mouse. (*b*) Representative micrographs of lung tissue using H/E staining. (*c*) Representative detail micrographs of H/E-stained lung tissue 72 h post-infection.

Having determined a non-physiologically high copy number of *exoY* in the PA103 recombinants that have been used to explore the pathogenicity of ExoY in infection models, we next compared the course of an acute airway infection in C57BL/6 J mice that received 10^6^ cfu of either B420, PT22, ExoY, ExoY^K81M^ or ΔExoY bacteria by intratracheal instillation. All mice experienced a loss of body weight and a drop of rectal temperature within the first few hours and developed clinical signs of disease, but the recipients of B420, PT22, ExoY^K81M^ or ΔExoY bacteria recovered thereafter (figure 2*a*). The ExoY recipients, however, continuously deteriorated during the observation period of 72 h. Lung histology revealed a similar outcome (figure 2*b*). Twelve hours after the instillation of bacteria inflammatory cells had emigrated into the lungs of all mice irrespective of the inoculated *P. aeruginosa* strain. By 72 h the number of inflammatory cells had declined in recipients of B420, PT22, ExoY^K81M^ or ΔExoY bacteria, whereas cellular infiltration and inflammation had increased in mice which had received the ExoY recombinant strain. These data demonstrate that the absence or presence of a single T3SS operon did not significantly affect the course of the acute airways infection in our murine model, but that a high copy number of plasmid-borne *exoY* despite the absence of *exoU* and *exoT* is sufficient to induce a substantially more severe course of local and systemic infection.

### 3.3. Concentrations of cNMPs in lung tissue and serum of infected mice

**Figure 3.**
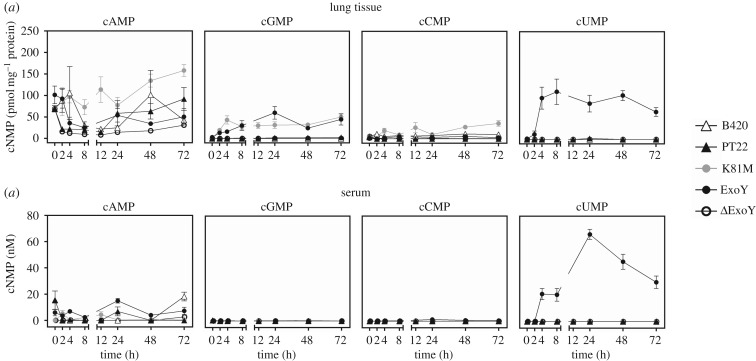
Concentrations of cNMPs in lung tissue and serum of infected mice. Lung tissue and serum were prepared and then processed for HPLC-MS/MS analysis as described in Material and methods. (*a*) Data shown represent concentration of cNMPs in lung tissue referred to protein concentration 0–72 h after infection with 10^6^ cfu per mouse. (*b*) Data shown represent accumulated serum concentrations of cNMPs 0–72 h after infection with 10^6^ cfu per mouse. (*a*,*b*) Data shown are the means ± s.e.m. of *n* = 6 animals. Note: baseline curves overlap.

ExoY is a promiscuous nucleotidyl cylase that synthesizes numerous cNMPs including the previously undescribed cUMP. We measured cNMP concentrations in lung tissue and serum during the acute murine airway infection with *P. aeruginosa.* Fluctuating levels of cAMP were recorded in all mice demonstrating that the production of cAMP was not influenced by the absence or presence of a T3SS operon or of a functional ExoY (figure 3). Some cGMP and cCMP were detectable in lungs of ExoY^K81M^ recipients ruling out that these cyclic nucleotides had been synthesized by ExoY. By contrast, high cUMP levels in both lungs and sera were exclusively measured in samples from mice that had been infected with the *P. aeruginosa* carrier of the *exoY-*plasmid. Thus neither the murine host nor *P. aeruginosa* chromosome-derived gene products but plasmid-borne ExoY had synthesized cUMP in the infected animals.

## 4. Discussion

The ExoY-overexpressing recombinant *P. aeruginosa* strains ExoY and its loss-of-function control ExoY^K81M^ have been used as informative tools to resolve the action of the exotoxin on mammalian cells *in vitro* and *in vivo*. Thereby ExoY was identified to be a promiscuous cyclase that synthesizes preferentially cUMP and cGMP *in vitro* [14], and mainly cUMP *in vivo* [15]. ExoY intoxication has been shown to hinder vascular repair following infection [11], to induce intercellular gap formation and to stimulate endothelial cell tau hyperphosphorylation and insolubility [10,11,13]. Hence ExoY may drive a proteinopathy of the endothelium in the infected host [13].

The outcome of this study does not contradict these findings on the action of the exotoxin ExoY. However, our data demonstrate that the recombinant PA103 strain is strongly overexpressing ExoY thanks to the presence of multiple copies of *exoY* in extrachromosomal plasmids. The engineered ExoY strain caused substantial morbidity and pathology in our murine infection model, but no difference in phenotype was seen between the ExoY-positive PT22, the ExoY negative B420, the ExoY knock-out ExoY^K81M^ and the vector-negative control ΔExoY. Our findings demonstrate that the reported [10,11,13] severe infectious phenotypes are caused by multi-copy plasmid-borne *exoY*. Thus the role of ExoY during infection with real-life *P. aeruginosa* remains elusive. ExoY may indeed be an exotoxin that stimulates an infectious proteinopathy, but up to now this phenotype has not been detected by the commonly applied infection models with natural *P. aeruginosa* strains. However, it must be kept in mind that these infection models focus on the role of ExoY in acute infections, whereas chronic infection models may uncover some specific ExoY-associated pathology. But at present we would like to conclude that earlier statements are still valid—that wild-type concentrations of ExoY ‘have little effect on virulence and cytotoxicity’ [8]. It remains to be seen whether the F-actin mediated stimulation of ExoY activity observed *in vitro* [28] under certain conditions translates *in vivo*.

## Data accessibility

All relevant data are presented in the figures in the manuscript.
